# Conserved determinants of lentiviral genome dimerization

**DOI:** 10.1186/s12977-015-0209-x

**Published:** 2015-09-29

**Authors:** Thao Tran, Yuanyuan Liu, Jan Marchant, Sarah Monti, Michelle Seu, Jessica Zaki, Ae Lim Yang, Jennifer Bohn, Venkateswaran Ramakrishnan, Rashmi Singh, Mateo Hernandez, Alexander Vega, Michael F. Summers

**Affiliations:** Howard Hughes Medical Institute, Department of Chemistry and Biochemistry, University of Maryland Baltimore County, 1000 Hilltop Circle, Baltimore, MD 21250 USA

**Keywords:** HIV-1_NL4-3_, HIV-2_ROD_, SIV_cpzTAN1_, SIV_cpzUS_, Retroviral genome dimerization, RNA structure, Labile dimer, Non-labile dimer, Gel electrophoresis, Nucleocapsid protein (NC)

## Abstract

**Background:**

Retroviruses selectively package two copies of their unspliced genomes by what appears to be a dimerization-dependent RNA packaging mechanism. Dimerization of human immunodeficiency virus Type-1 (HIV-1) genomes is initiated by “kissing” interactions between GC-rich palindromic loop residues of a conserved hairpin (DIS), and is indirectly promoted by long-range base pairing between residues overlapping the *gag* start codon (AUG) and an upstream Unique 5′ element (U5). The DIS and U5:AUG structures are phylogenetically conserved among divergent retroviruses, suggesting conserved functions. However, some studies suggest that the DIS of HIV-2 does not participate in dimerization, and that U5:AUG pairing inhibits, rather than promotes, genome dimerization. We prepared RNAs corresponding to native and mutant forms of the 5′ leaders of HIV-1 (NL4-3 strain), HIV-2 (ROD strain), and two divergent strains of simian immunodeficiency virus (SIV; cpz-TAN1 and -US strains), and probed for potential roles of the DIS and U5:AUG base pairing on intrinsic and NC-dependent dimerization by mutagenesis, gel electrophoresis, and NMR spectroscopy.

**Results:**

Dimeric forms of the native HIV-2 and SIV leaders were only detectable using running buffers that contained Mg^2+^, indicating that these dimers are more labile than that of the HIV-1 leader. Mutations designed to promote U5:AUG base pairing promoted dimerization of the HIV-2 and SIV RNAs, whereas mutations that prevented U5:AUG pairing inhibited dimerization. Chimeric HIV-2 and SIV leader RNAs containing the dimer-promoting loop of HIV-1 (DIS) exhibited HIV-1 leader-like dimerization properties, whereas an HIV-1_NL4-3_ mutant containing the SIV_cpzTAN1_ DIS loop behaved like the SIV_cpzTAN1_ leader. The cognate NC proteins exhibited varying abilities to promote dimerization of the retroviral leader RNAs, but none were able to convert labile dimers to non-labile dimers.

**Conclusions:**

The finding that U5:AUG formation promotes dimerization of the full-length HIV-1, HIV-2, SIV_cpzUS_, and SIV_cpzTAN1_ 5′ leaders suggests that these retroviruses utilize a common RNA structural switch mechanism to modulate function. Differences in native and NC-dependent dimerization propensity and lability are due to variations in the compositions of the DIS loop residues rather than other sequences within the leader RNAs. Although NC is a well-known RNA chaperone, its role in dimerization has the hallmarks of a classical riboswitch.

## Background

Retroviral RNA genomes participate in multiple activities during viral replication and do not simply function as passive carriers of genetic information. They are directly involved in transcriptional activation, splicing, frame shifting, dimerization, intracellular trafficking, genome selection and packaging, reverse transcription, and stabilization of the structure of the mature virion [1]. Although current understanding of the mechanisms that regulate these activities is incomplete, studies indicate that several activities are temporally modulated by conformational changes within the viral genome. In particular, dimerization of the human immunodeficiency virus type-1 (HIV-1) genome appears to serve as a functional switch that down-regulates splicing and translational activity and promotes recruitment of the unspliced, pseudo-diploid genome into assembling virions [2–19].

**Fig. 1:**
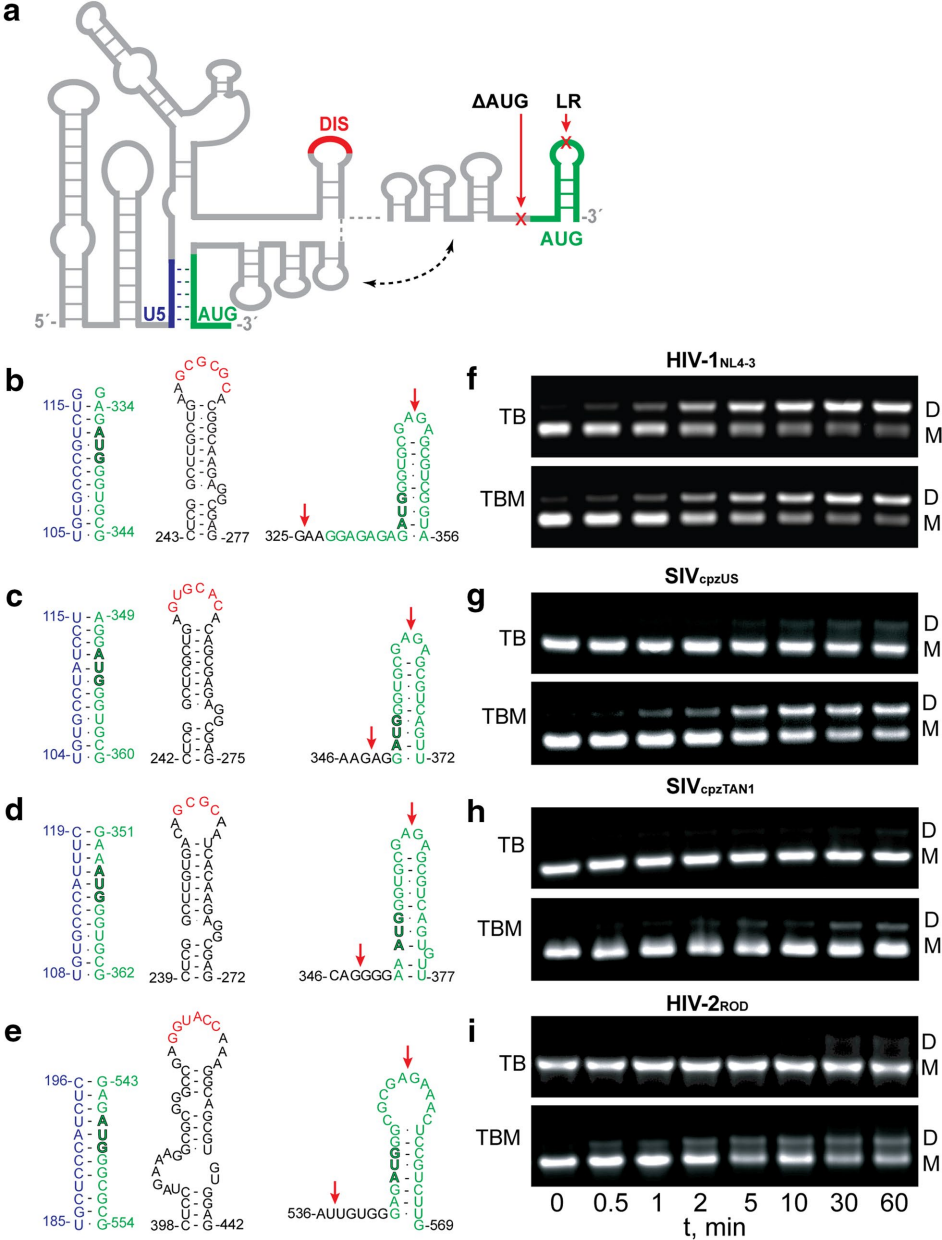
Essential elements involved in retroviral 5′-leader dimerization. **a** Representation of retroviral 5′-leader showing AUG (*green*) in hairpin and U5:AUG conformations. *Arrows* indicate locations of 3′ truncations. Nucleotide sequences and predicted U5:AUG base pairing, DIS with palindromic region (*red*), and AUG hairpins of **b** HIV-1_NL4-3_, **c** SIV_cpzUS_, **d** SIV_cpzTAN1_, and **e** HIV-2_ROD_. The U5:AUG formation, DIS, and AUG hairpins for HIV-1_NL4-3_ 5′-leader dimerization have been proposed previously [33]. **f**–**i** Native gel electrophoresis data obtained for each retroviral full-length leader (5′-L_WT_) after time-dependent incubation in PI buffer and detected using TB (*top panel*) and TBM (*lower panel*) running buffers. Positions of the monomer and dimer bands are denoted by M and D, respectively. 5′-L_WT_ constructs reached equilibrium within 30-min to 1-h incubation time

Many of the diverse functions of retroviral genomes are promoted or regulated by elements located within the 5′ leader, the most conserved region of the viral RNA [1, 20]. Combinations of site-directed mutagenesis, chemical and enzymatic accessibility experiments, phylogenetic studies, and free energy calculations indicate that the 5′ leader of HIV-1 consists of a series of stem-loop structures connected by relatively short linkers [13, 21–30]. Early in vitro nucleotide reactivity experiments also indicated that the secondary structure changes upon dimerization [31–34], and a phylogenetic analysis of primate lentiviruses led to proposals that the dimeric conformer is stabilized by long-range base pairing between residues overlapping the *gag* start codon (AUG) and an upstream Unique 5′ element (U5), Fig. 1 [13]. U5:AUG complementarity was also observed in evolutionarily distant retroviruses [27], and it was suggested that U5:AUG formation may be coupled with conformational changes that influence the functional properties of the RNA [13, 27, 35]. NMR studies confirmed the presence of U5:AUG base pairing in the dimeric form of the HIV-1 leader RNA [33], and U5:AUG formation was further shown to promote both dimerization and binding to the cognate nucleocapsid (NC) protein in vitro, as well as selective and efficient packaging of vector RNAs into virus-like particles in transfected cell cultures [33]. These and other findings suggested packaging mechanisms in which a U5:AUG dependent RNA structural switch leads to the formation of structures that expose both the dimer initiation site (DIS) and high affinity NC binding sites, thereby promoting selective packaging of the dimeric genome [2, 13, 21, 33, 36].

The conservation of U5:AUG sequence complementarity [13, 27] suggests that the U5:AUG dependent dimerization mechanism proposed for HIV-1 might also be utilized by other retroviruses (Fig. 1). Three related but evolutionarily divergent lentiviruses have been studied previously and provide suitable comparisons: the human immunodeficiency virus Type-2 (HIV-2), the simian immunodeficiency virus isolated from a chimpanzee raised in the U.S. (SIV_cpzUS_) [37] that is closely related to HIV-1 [38], and an evolutionarily divergent SIV isolated from a chimpanzee in Tanzania [39] (SIV_cpzTAN1_). The 5′ leaders of all three retroviruses have been predicted to adopt secondary structures with similarities to that observed for the HIV-1_NL4-3_ leader [40–49]. All of these retroviral leaders contain similarly located hairpins with palindromic loops (DIS; Fig. 1b–e) that are believed to function as a primary dimer initiation site [7, 14, 21, 33, 41, 42], and all also exhibit U5:AUG sequence complementarity, Fig. 1 [13, 27]. Despite these similarities, the in vitro dimerization behavior of these leaders differs from that of HIV-1. For example, whereas the HIV-1 5′ leader forms a thermally stable “tight” dimer (herein called a “non-labile dimer”) that can be readily detected by native gel electrophoresis at ambient temperature using Tris–borate running buffers that lack Mg^2+^ (TB condition) [50], HIV-2 5′ leader constructs that extend at least through the 5′-half of the AUG element, and are thus capable of base pairing with U5, have been shown to form “loose” dimers (herein called “labile dimers”) that are only distinguishable from monomers on gels obtained at 4 °C with Mg^2+^ in the TB running buffer (TBM condition) [43, 50]. In addition, truncations that prevent U5:AUG base pairing were shown to promote the formation of non-labile HIV-2 RNA dimers [43, 44], suggesting that U5:AUG base pairing inhibits, rather than promotes, dimerization of the HIV-2 5′- leader [45, 46, 48] (a previously noted dichotomy [27]).

To explore the potential roles of the AUG hairpin, U5:AUG base pairing, the DIS palindromes, and the influence of NC on retroviral genome dimerization, we have conducted in vitro kinetic and thermodynamic dimerization studies with “full-length” HIV-1_NL4-3_, SIV_cpzTAN1_, SIV_cpzUS_, and HIV-2_ROD_ 5′ leader RNAs that include the entire AUG hairpin, and with mutants analogous to those employed in recent dimerization studies of the HIV-1 leader [33]. We also examined the influence of non-native 3′-residues, and pH variations, on dimer formation rates and the position of the monomer–dimer equilibrium.

## Results and discussion

### RNA construct design

Full-length, wild-type constructs (5′-L_WT_) for HIV-1_NL4-3_, SIV_cpzTAN1_, SIV_cpzUS_, and HIV-2_ROD_ viral strains were designed to include all residues required for the formation of the proposed 3′-terminal AUG (Fig. 1a) [33, 51]. Engineering of truncated constructs was guided by recent studies of the HIV-1_NL4-3_ leader [33]. Thus, one set of constructs lacked distal residues required for AUG hairpin formation (but allowing U5:AUG pairing; 5′-L_LR_) and a second set of 5′-L RNAs lacked all residues required for both AUG hairpin formation at U5:AUG pairing (see arrows in Fig. 1b–e) [33]. A third set of constructs was engineered to test the role of the DIS loop residues on the dimerization properties of the SIV_cpzTAN1_ and HIV-2_ROD_ 5′-L constructs (see below). All of these RNAs were engineered to contain native residues at the 3′ termini (A356, U372, U377, and G569 for wild type HIV-1_NL4-3_, SIV_cpzUS_, SIV_cpzTAN1_, and HIV-2_ROD_ 5′-L constructs, respectively) to avoid potential influences of non-native 3′-residues (utilized in prior studies for DNA template linearization [33, 52]) on dimerization (see below).

### Non-native 3′-residues influence the dimerization behavior of the HIV-1 5′ leader

Time-dependent RNA dimerization data were obtained by dissolution of a concentrated buffer/salt solution into HIV-1_NL4-3_ 5′-L_WT_ solutions that were pre-incubated at low ionic strength, such that the final solutions contained physiological-like ionic conditions (PI buffer; 140 mM KCl, 10 mM NaCl, 1 mM MgCl_2_, 10 mM Tris–HCl, pH 7.0). At low ionic strength, a single band corresponding to the monomeric HIV-1_NL4-3_ 5′-L_WT_ RNA was observed, Fig. 1f. Time dependent appearance of a band corresponding to the dimeric species was observed upon adjustment to PI conditions, and the system reached equilibrium after approximately 1 h of incubation time, Fig. 1f. Essentially identical results were obtained using TB and TBM running buffers, Fig. 1f. Similar results were also reported for RNAs corresponding to the full-length 5′ leader of the LAI strain of HIV-1 [53], and the dimeric form of the HIV-1_NL4-3_ 5′ leader has thus been referred to as a “tight” (kinetically non-labile) dimer [39, 54, 55]. Interestingly, the dimerization rate is faster (less than one hour to reach equilibrium, compared to ~24 h in previous studies), and the dissociation equilibrium constant smaller (~2-fold; Kd = 0.40 ± 0.13 μM), than values observed previously in our laboratory for an RNA construct that contains three additional 3′-cytosines [33]. We attribute these differences to the ability of the additional non-native residues to artificially stabilize the AUG hairpin, thereby favoring the monomeric form of the non-native RNA.

### The HIV-2_ROD_, SIV_cpzUS_, and SIV_cpzTAN1_ 5′ leaders form dimers that are thermo-dynamically stable but kinetically labile

Time-dependent RNA dimerization data were obtained similarly for SIV_cpzUS_, SIV_cpzTAN1_, and HIV-2_ROD_ 5′-L_WT_ constructs. Equilibrium was achieved after ~30-min incubation in PI buffer (Fig. 1f–i). Unlike HIV-1_NL4-3_ 5′-L_WT_, the dimeric forms of SIV_cpz_ and HIV-2_ROD_ 5′-L_WT_ were only detectable on gels using TBM as the running buffer. Similar results were previously reported for a HIV-2_ROD_ 5′ leader construct that terminates near the center of the AUG hairpin (residues 1–561) [43–46]. The inability to detect significant amounts of a dimer species using TB as the running buffer is likely due to the rapid intrinsic dissociation rate of the dimeric RNAs and the dependence on Mg^2+^ for dimer stability. In other words, it is possible that, as the RNA migrates toward the anode, the Mg^2+^ ions migrate toward the cathode and away from the RNA, resulting in a rapid dimer to monomer conversion. Without Mg^2+^ in the running buffer (TB conditions), the RNA migrates predominantly or exclusively as a monomer, whereas the presence of Mg^2+^ under TBM conditions prevents or limits the extent of electrophoretic-dependent dimer dissociation. Thus, the SIV_cpzUS_, SIV_cpzTAN1_, and HIV-2_ROD_ 5′ leader RNAs form dimeric species that are thermodynamically stable but kinetically labile.

### Effects of AUG truncations and deletions on dimer formation

**Fig. 2:**
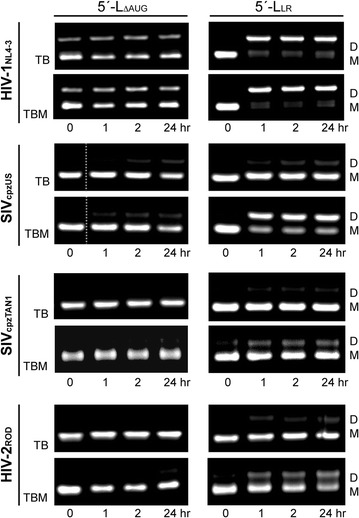
The monomer–dimer equilibrium of retroviral 3′-deleted leader constructs. Native agarose gel electrophoresis data showing that HIV-1_NL4-3_, SIV_cpzUS_, SIV_cpzTAN1_, and HIV-2_ROD_ 5′-L_ΔAUG_ and 5′-L_LR_ constructs incubated for 0, 1, 2 and 24 h in PI buffer (removal of t = 30 min lanes in SIV_cpsUS_ panels denoted by *dashed lines*). Monomer:dimer equilibrium is reached within 1 h of incubation for all constructs

**Fig. 3:**
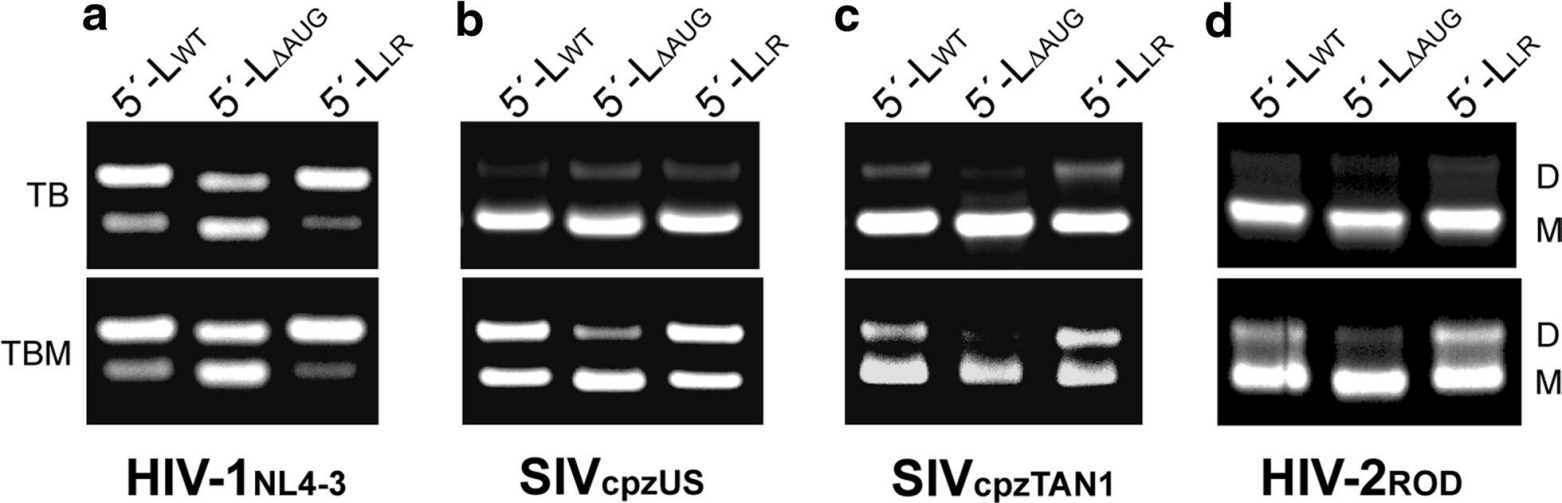
The U5:AUG formation promotes retroviral 5′-leader dimerization. Native agarose gel electrophoresis data obtained for leader constructs (5′-L_WT_, 5′-L_ΔAUG_, and 5′-L_LR_) of **a** HIV-1_NL4-3_, **b** SIV_cpzUS_, **c** SIV_cpzTAN1_, and **d** HIV-2_ROD_ [after 1-h incubation in PI buffer and measured using TB (*top panel*) and TBM (*lower panel*) as the running buffer]. Monomer and dimer bands are denoted as M and D, respectively. As observed for analogous HIV-1_NL4-3_ leader constructs, truncations that inhibit U5:AUG formation (5′-L_ΔAUG_) also inhibit dimerization, whereas those designed to favor U5:AUG formation (5′-L_LR_) promote or restore dimerization. Quantitative analysis of gels [56] afforded the following % dimer values (reported as mean ± standard deviation) for two TBM gels for 5′-L_WT_; 5′-L_ΔAUG_; 5′-L_LR_: **a** 52 ± 3; 32 ± 2; 64 ± 5. **b** 29 ± 5; 17 ± 3; 34 ± 5. **c** 18 ± 1; 5 ± 1; 21 ± 5. **d** 14 ± 3; 5 ± 2; 20 ± 3

To determine the potential effects of U5:AUG base pairing on dimerization, the equilibrium behaviors of the full-length and 3′-deletion constructs were analyzed by native agarose gel electrophoresis. For all constructs, equilibrium was reached after less than 1 h of incubation in PI buffer, Fig. 2. Significant differences in monomer:dimer band intensities were observed under TBM running condition for each viral strain, Fig. 3. For all viral strains, the 5′-L_∆AUG_ construct showed decreased dimerization compared to the 5′-L_WT_. For SIV_cpzUS_, SIV_cpzTAN1_, and HIV-2_ROD_, the 5′-L_LR_ and 5′-L_WT_ gave rise to similar amounts of dimer (Fig. 3b–d), while the HIV-1_NL4-3_ 5′-L_LR_ gave rise to similar or greater amounts of dimer than the cognate 5′-L_WT_, Fig. 3a. These relative dimerization propensities (5′-L_LR_ ≥ 5′-L_WT_ > 5′-L_∆AUG_) suggest that the 5′-most residues of the AUG region play a critical role in the 5′ leader dimerization, in which deletion of the entire AUG essentially inhibits dimerization whereas deletion of only the 3′-most residues of the AUG stem-loop promotes or restores dimerization. The effects of AUG truncations and deletions on 5′-L RNAs are consistent with previous studies of HIV-1_NL4-3_ leader RNAs [33].

### Influence of pH on dimerization

**Fig. 4:**
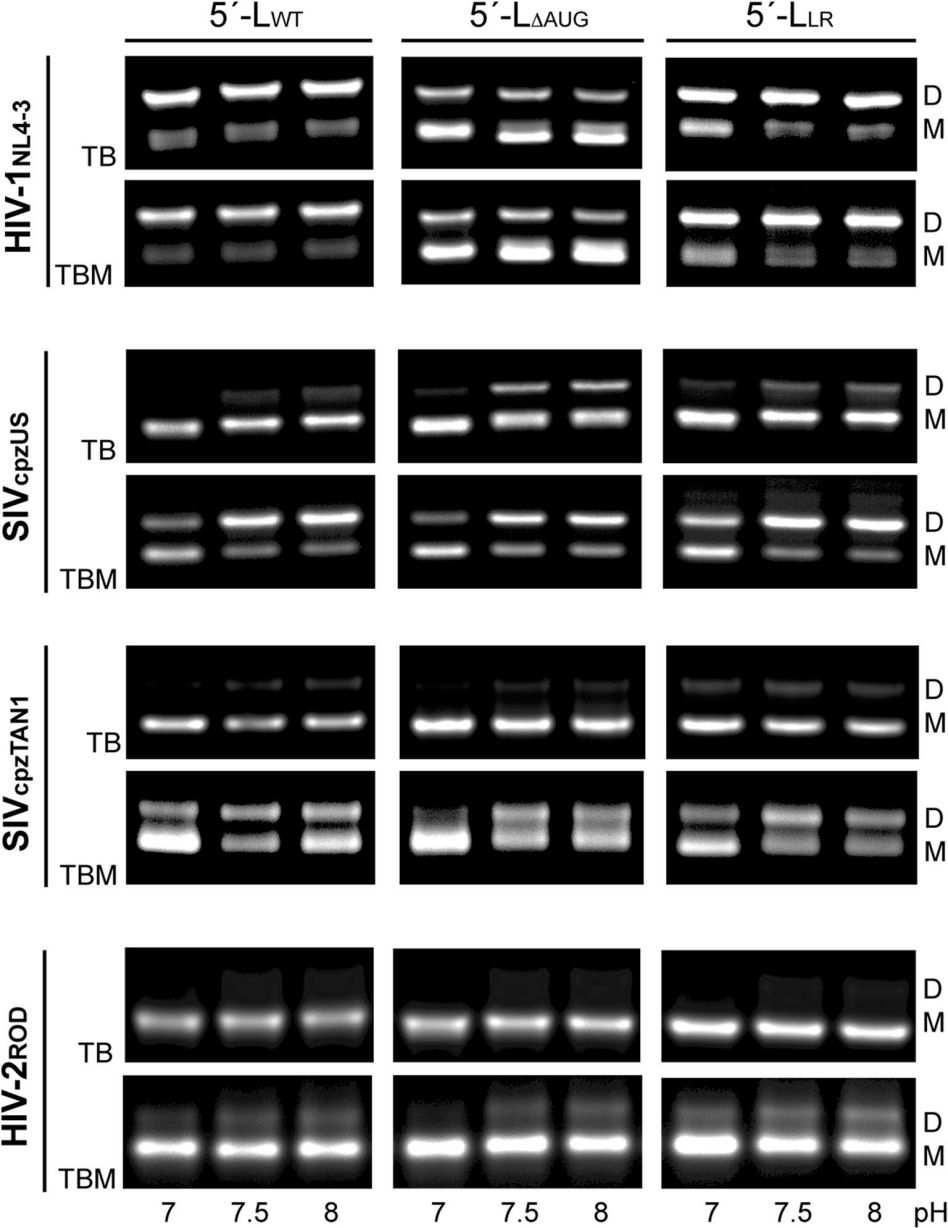
pH dependent dynamics of HIV-1_NL4-3_, SIV_cpzUS_, SIV_cpzTAN1_, and HIV-2_ROD_ 5′-leader dimerization. Each retroviral 5′-L_WT_, 5′-L_ΔAUG_, and 5′-L_LR_ constructs were incubated for 1 h in buffers containing PI salt and an indicated pH range. The monomers (M) and dimers (D) in native agarose gels are indicated. SIV_cpzUS_ and SIV_cpzTAN1_ leader dimerization is promoted by the increase of pH, whereas pH with 7.0–8.0 has no significant effect on HIV-1_NL4-3_ and HIV-2_ROD_ leader dimerization

To investigate the influence of pH on the dimerization, we incubated 5′-L_WT_, 5′-L_ΔAUG_, and 5′-L_LR_ RNAs of each viral strain in buffers containing PI salts and pH values in the range of 7.0–8.0 and measured dimerization by native agarose gel electrophoresis, Fig. 4. The HIV-1_NL4-3_ RNA constructs were insensitive to the changes in pH, as measured using either TB or TBM running buffers. The HIV-2_ROD_ constructs were also relatively insensitive to changes in pH, although minor dimer bands were detectable at higher pH values for the 5′-L_LR_ construct, Fig. 4. However, the SIV_cpzUS_ RNAs exhibited a dramatic equilibrium shift from a primarily monomeric species at pH 7.0 to a primarily dimeric species at pH 8.0, Fig. 4. The SIV_cpzTAN1_ constructs also exhibited significant equilibrium shifts toward the dimeric species at the higher pH values, Fig. 4. Although RNA structure and dynamics can be influenced by changes in pH, our analysis of predicted base pairing patterns of the monomeric and dimeric forms of these RNAs did not lead to an explanation for the significant differences in pH dependence (e.g., scanning for potential A^+^·C mismatches that might differentially favor the monomer).

### NMR evidence for U5:AUG base pairing

**Fig. 5:**
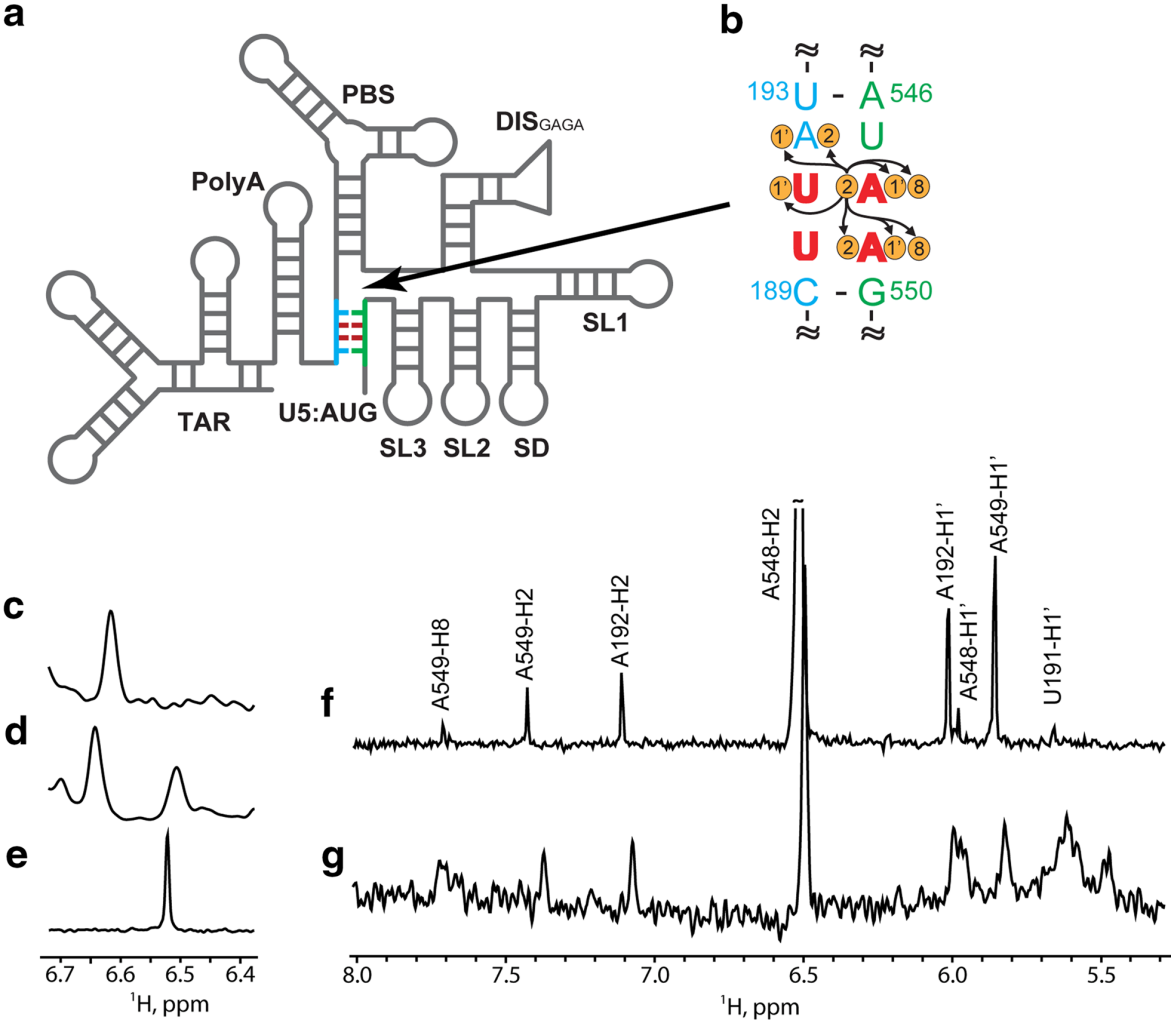
NMR detection for HIV-2_ROD_ U5:AUG formation. **a** Schematic representation of the dimeric HIV-2 5′-leader showing DIS exposure and long range base pairing between the U5 (*cyan*) and AUG (*green*) elements. **b** Portion of the lr-AID modified U5:AUG helix showing C-G to U-A substitutions in *red*. **c**–**e** Portions of the 1D ^1^H NMR spectra obtained for HIV-2_ROD_ 5′-L, lr-AID substituted HIV-2_ROD_ 5′-L_GAGA_, and a control oligo-RNA containing the lr-AID substituted U5:AUG stem, respectively. Signals at ~6.51 ppm are due to the A548-H2 (lr-AID) signal. **f** F2 slice from the 2D NOESY spectrum obtained for the control lr-AID U5:AUG oligonucleotide. Signal assignments were made by standard connectivity methods [57]. **g** 1D saturation-difference NOE spectrum obtained for lr-AID substituted HIV-2_ROD_ 5′-L_GAGA_. Except for expected intensity differences (due to differences in rotational correlation time and NOE mixing periods employed), the spectral features are similar to those of the control RNA, indicating that both RNAs adopt the predicted U5:AUG helix

The above studies provide indirect evidence that dimer formation is promoted by U5:AUG base pairing. To directly probe for U5:AUG base pairing, we employed a recently developed NMR-based approach that takes advantage of the unique NMR chemical shift patterns of adenosine H2 protons when incorporated into rarely observed [UUA]:[AAU] base pair triplets (called long-range Adenosine Interaction Detection, lr-AID) [33]. The approach is similar to a FRET experiment in that it provides local distance information, but differs in that it is dependent on conservative base pair substitution for signal detection rather than chemical modification and affords both structural (from the chemical shifts) and distance (from the nuclear Overhauser effect, NOE) information over a shorter distance range (typically <5 Å). We substituted the GC-rich DIS loop residues (^418^AGGUACCAAA^428^) by a GAGA tetraloop (HIV-2_ROD_ 5′-L_GAGA_) to reduce the size of the RNA and thereby enhance NMR signal detection, without affecting the predicted secondary structure of the RNA (an approach employed previously in studies of the HIV-1 5′-leader [36, 52]). The HIV-2_ROD_ 5′-L_GAGA_ RNA construct was prepared in which two sequential C-G base pairs in the U5:AUG helix were substituted by A-U pairs, Fig. 5a, b, and for comparison, a model U5:AUG oligonucleotide RNA containing the same C-G to U-A substitutions was prepared. As shown in Fig. 5, the lr-AID substituted 5′-L RNA gave rise to the expected upfield-shifted A548-H2 NMR signal (6.51 ppm), which occurred in a region of the spectrum that was free of signals in the spectra obtained for 5′-L RNAs containing fully native U5 and AUG sequences (Fig. 5c, d) and was similar to the A548-H2 signal observed for the control oligoribonucleotide (Fig. 5e). NOEs and chemical shifts of the interacting cross-strand and sequential protons observed in a one-dimensional saturation-difference NOE spectrum were similar to those observed for the control RNA, Fig. 5f, g, indicating that both RNAs contain the predicted U5:AUG helix.

### Dimerization is mediated by the DIS loop

**Fig. 6:**
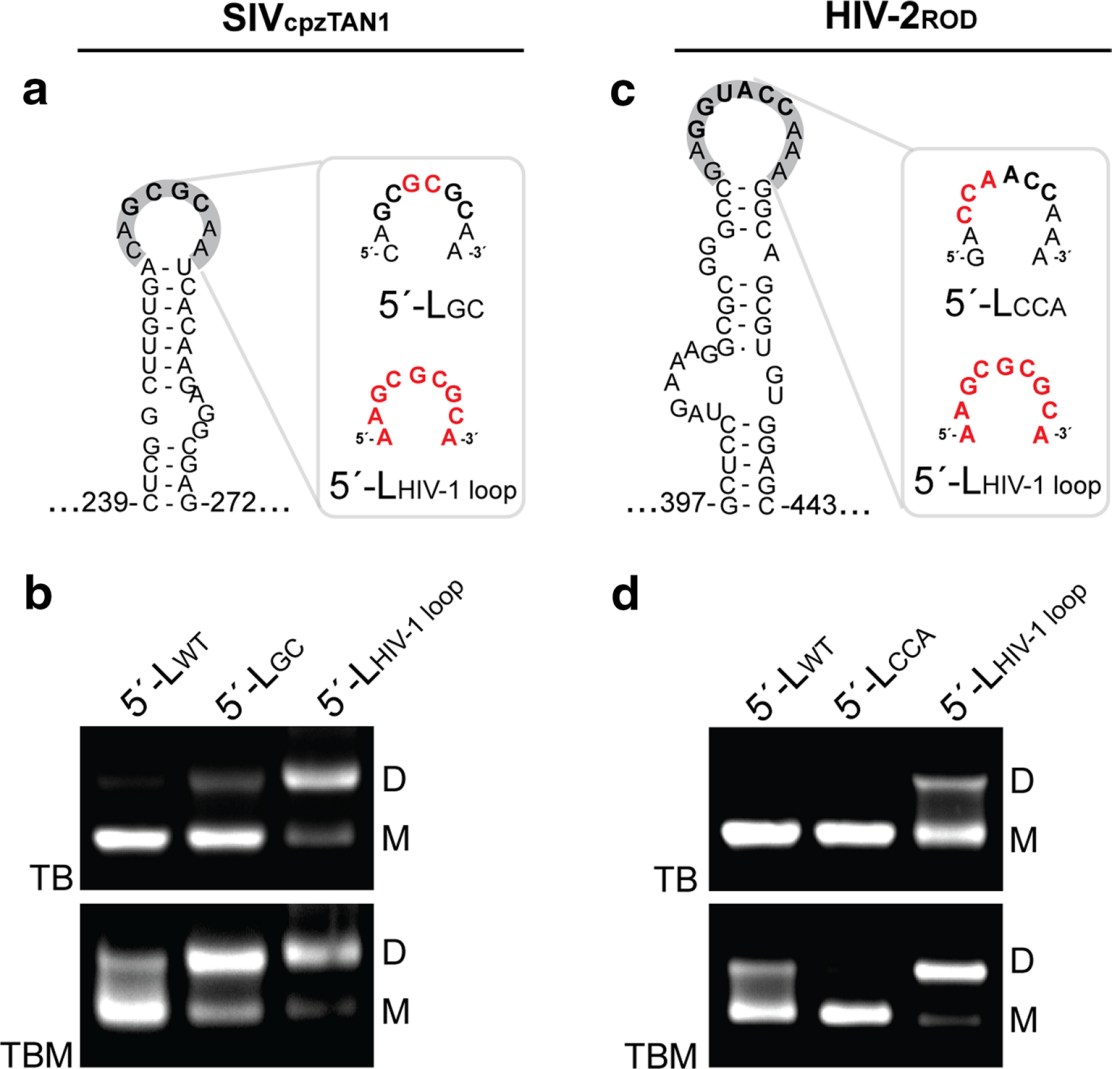
DIS mutations affect retroviral 5′-leader dimerization. **a** Structure of the native SIV_cpzTAN1_ DIS stem-loop within the intact leader and mutations (*red*) that extend its native DIS palindrome. **b** Native agarose gel electrophoresis data obtained for SIV_cpzTAN1_ wild-type (5′-L_WT_) and mutant leader RNAs (5′-L_GC_ and 5′-L_HIV-1 loop_) after 1-h incubation in PI buffer and measured using TB and TBM running buffers. **c** Structure of the native HIV-2_ROD_ DIS stem-loop within the intact leader and mutations (*red*) that obstructs the DIS palindrome (5′-L_CCA_) or enhances DIS intermolecular loop–loop interactions (5′-L_HIV-1 loop_). **d** Native gel data also obtained for HIV-2_ROD_ wild-type and mutant leader RNAs. DIS mutations with extended/enhanced DIS palindromes favor the dimer formation, whereas mutation with obstructed DIS palindrome favors the monomer formation

To better understand the factors that influence dimer lability, we determined the dimerization properties of SIV_cpzTAN1_ and HIV-2_ROD_ 5′-L RNAs that contained mutated DIS loops. The DIS loop of SIV_cpzTAN1_ contains a four-residue palindrome (GCGC) and is thus predicted to form a four base-pair kissing intermolecular interface. Substitution of the GCGC palindrome by a 6-residue GCGCGC palindrome (as contained in the HIV-1_NL4-3_ loop) resulted in an equilibrium that included a significant amount of labile dimer and some non-labile dimer (as detected using TBM and TB running buffers, respectively), Fig. 6a, b. Interestingly, substitution of the entire SIV_cpzTAN1_ loop (^251^CAGCGCAA^258^) by the intact HIV-1_NL4-3_ loop (AAGCGCGCA) resulted in an equilibrium very similar to that observed for the native HIV-1_ROD_ leader, in which the RNA exists predominantly as a non-labile dimer, Fig. 6a, b. Similarly, substitution of the DIS loop residues of HIV-2_ROD_ (^418^GAGGUACCAAA^428^) by the intact HIV-1_NL4-3_ DIS loop (AAGCGCGCA) led to dimerization behavior essentially matching that observed for the native HIV-1_NL4-3_ 5′ leader, Fig. 6c, d. We also mutated the first three bases in the HIV-2_ROD_ DIS palindrome (^420^GGU^422^) to CCA, a construct previously engineered to prevent intermolecular DIS-mediated kissing interactions [49], Fig. 6c. Whereas the native HIV-2_ROD_ 5′ leader RNA forms readily detected non-labile dimers, and the chimeric HIV-2_ROD_ RNA containing the HIV-1_NL4-3_ DIS loop forms non-labile dimers, no dimers (labile or non-labile) were detected for the HIV-2_ROD_ mutant that lacks a palindromic DIS loop, Fig. 6d.

### NC promotes dimerization, but does not convert labile to non-labile dimers

**Fig. 7:**
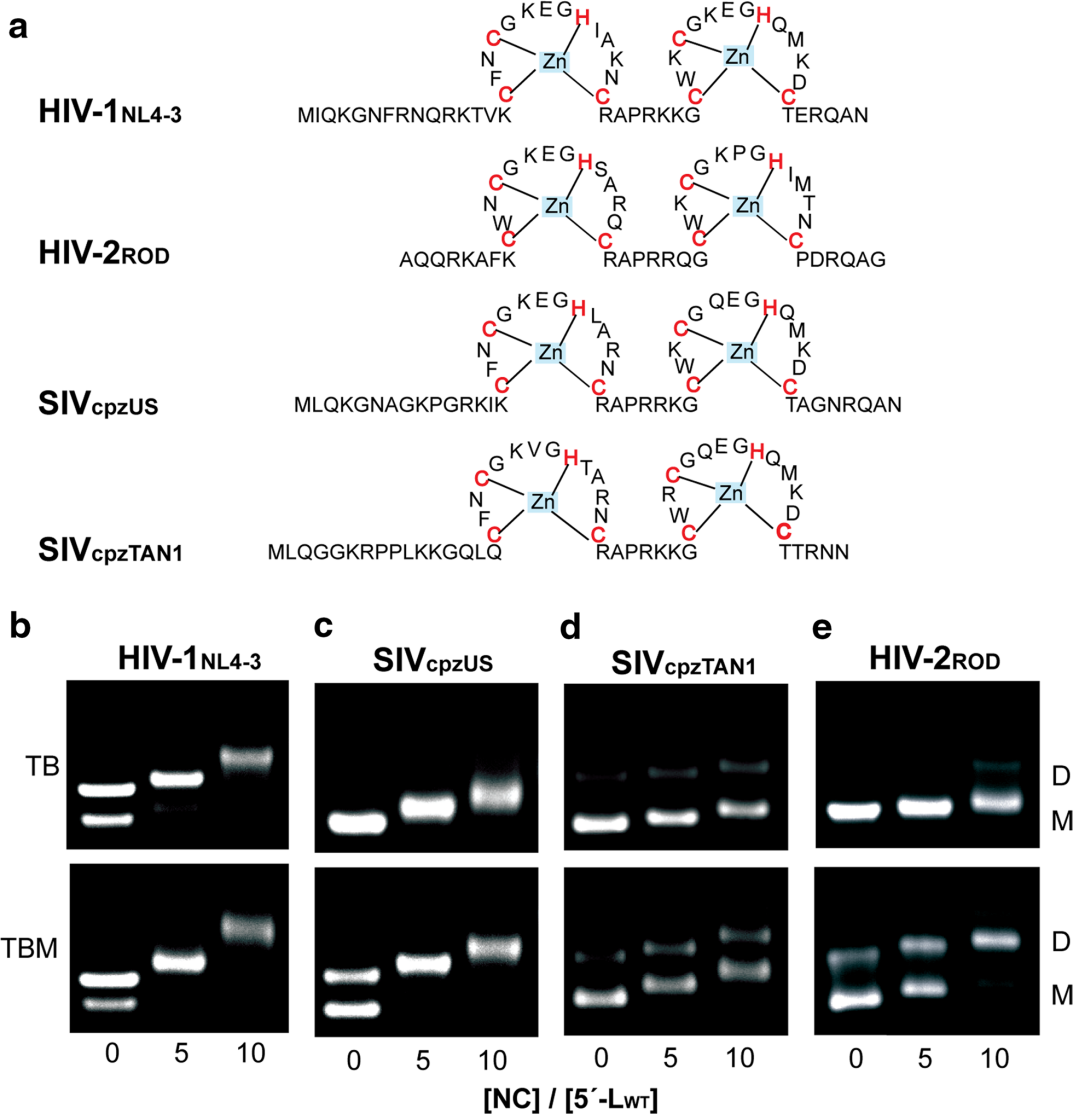
NC binding promotes dimer formation. **a** Secondary structures of NC proteins for HIV-1_NL4-3_, SIV_cpzUS_, SIV_cpzTAN1_, and HIV-2_ROD_. The zinc fingers motifs (CCHC, *red*) are strictly conserved among selected viral strains. **b**–**e** Native agarose gel NC titration results obtained for HIV-1_NL4-3_, SIV_cpzUS_, SIV_cpzTAN1_, and HIV-2_ROD_ 5′-L_WT_ in PI buffer. Addition of 0, 5, 10 equivalents of NC to the respective retroviral 5′-leader RNAs was probed using TB and TBM running buffers. Monomers (M) and dimers (D) are indicated

Retroviral NC proteins can function as both high-affinity RNA binding domains [58–62] and potent RNA chaperones [55, 63–67]. Previous studies have shown that the HIV-1_NL4-3_ NC protein can readily facilitate conversion of a large fragment of the HIV-1_NL4-3_ 5′ leader RNA (nucleotides 72–402) from a predominantly monomeric state to a predominantly dimeric (non-labile) state [55]. To determine if the SIV and HIV-2 NC proteins behave similarly, we cloned, expressed and purified the cognate NC proteins (Fig. 7a) and examined their effects on 5′-L dimerization. Full-length HIV-1_NL4-3_, SIV_cpzTAN1_, SIV_cpzUS_, and HIV-2_ROD_ 5′-L_WT_ RNAs were incubated in PI buffer containing increasing amounts of the respective NC proteins (NC:RNA = 0:1 to 10:1; 1 h incubation time), and the effects on dimerization were assayed by electrophoresis under TB and TBM running conditions. HIV-1_NL4-3_ NC induced an equilibrium shift of the HIV-1 5′-L_WT_ RNA that strongly favored the dimer at NC:RNA stoichiometries as low as 5:1, Fig. 7b. Similar results were obtained with the TB and TBM running buffers, indicating that, as observed for the HIV-1 5′-L_WT_ RNA in the absence of NC, the NC-induced dimers are non-labile, Fig. 7b. The addition of HIV-1_NL4-3_ NC also led to a slight retardation of the electrophoretic migration rates of the monomeric and dimeric species, indicating that HIV-1_NL4-3_ NC binds to both the monomeric and dimeric RNAs.

Incubation of the SIV_cpzUS_ and HIV-2_ROD_ 5′-L_WT_ RNAs with increasing amounts of the cognate NC proteins also resulted in significant equilibrium shifts toward the dimer as detected using the TBM running buffers. However, gels obtained for the same samples using TB as the running buffer did not contain significant dimer bands (Fig. 7c, e). All bands showed minor NC-dependent shifts. These findings indicate that NC is capable of binding to the SIV_cpzUS_ and HIV-2_ROD_ 5′-L_WT_ RNAs, and that binding induces an equilibrium shift that favors the labile dimeric species. Thus, dimers formed by the SIV_cpzUS_ and HIV-2_ROD_ 5′-L_WT_ RNAs are labile when formed either in the presence or absence of the cognate NC proteins. The SIV_cpzTAN1_ NC protein also induced band shifts indicative of its binding to the monomeric and dimeric forms of the SIV_cpzTAN1_ 5′-L_WT_ RNA, but in this case, the detected equilibrium shift toward the dimer was very small, Fig. 7d.

**Fig. 8:**
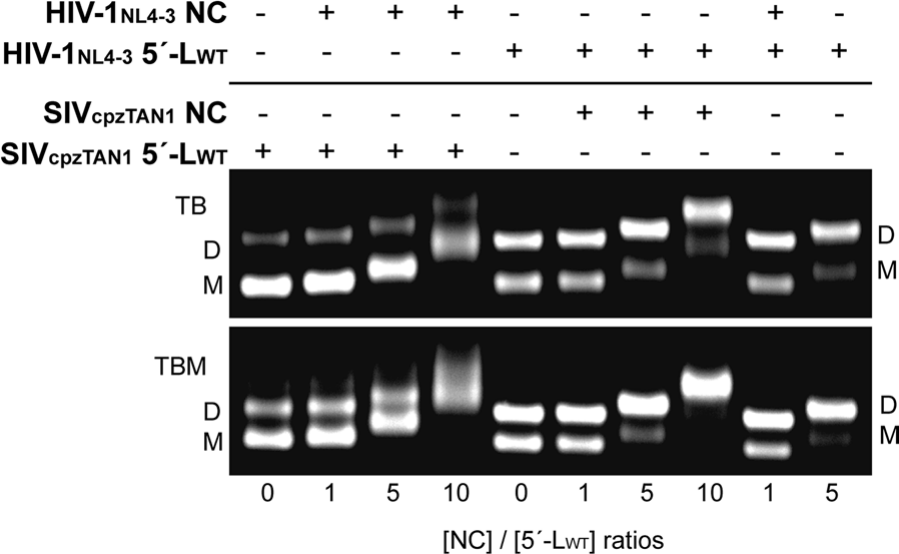
SIV_cpzTAN1_ NC can promote HIV-1_NL4-3_ 5′-L_WT_ in vitro dimerization. Native agarose gel under TB and TBM running conditions were employed to detect the viral RNA leader equilibrium shift from monomer to dimer as NC was titrated at 0:1, 1:1, 5:1, 10:1 molar ratios of [NC]:[RNA]. In the first four *lanes*, HIV-1_NL4-3_ NC was added into SIV_cpzTAN1_ 5′-L_WT_. In the next four *lanes*, SIV_cpzTAN1_ NC was added into HIV-1_NL4-3_ 5′-L_WT_. In the last two *lanes*, HIV-1_NL4-3_ NC was added into HIV-1_NL4-3_ 5′-L_WT_. The presence or absence of protein or RNA in the ribonucleoprotein mixture is denoted as + or − sign. The monomer (M) and dimer (D) bands are marked. Note that the mobility of the HIV-1 NC-shifted SIV_cpzTAN1_ 5′-L_WT_ monomer band (at 10:1 ratio) is similar to the mobility of the NC-free dimer band

The unexpected finding that HIV-1 NC strongly promotes dimerization of the cognate 5′-leader RNA, but SIV_cpzTAN1_ NC does not promote significant dimerization of the SIV_cpzTAN1_ RNA, led us to test whether these differential effects were due to the different properties of the NC proteins or the RNAs. As shown in Fig. 8, neither the HIV-1 _NL4-3_ nor the SIV_cpzTAN1_ NC proteins were able to induce significant dimerization of the SIV_cpzTAN1_ 5′-leader RNA. Conversely, the both the HIV-1_NL4-3_ and SIV_cpzTAN1_ NC proteins promoted dimerization of the HIV-1_NL4-3_ 5′-leader, Fig. 8. These findings indicate that the relative insensitivity of the SIV_cpzTAN1_ RNA to NC-dependent dimerization is due to the intrinsic property of the RNA and not to differences in the cognate NC protein.

**Fig. 9:**
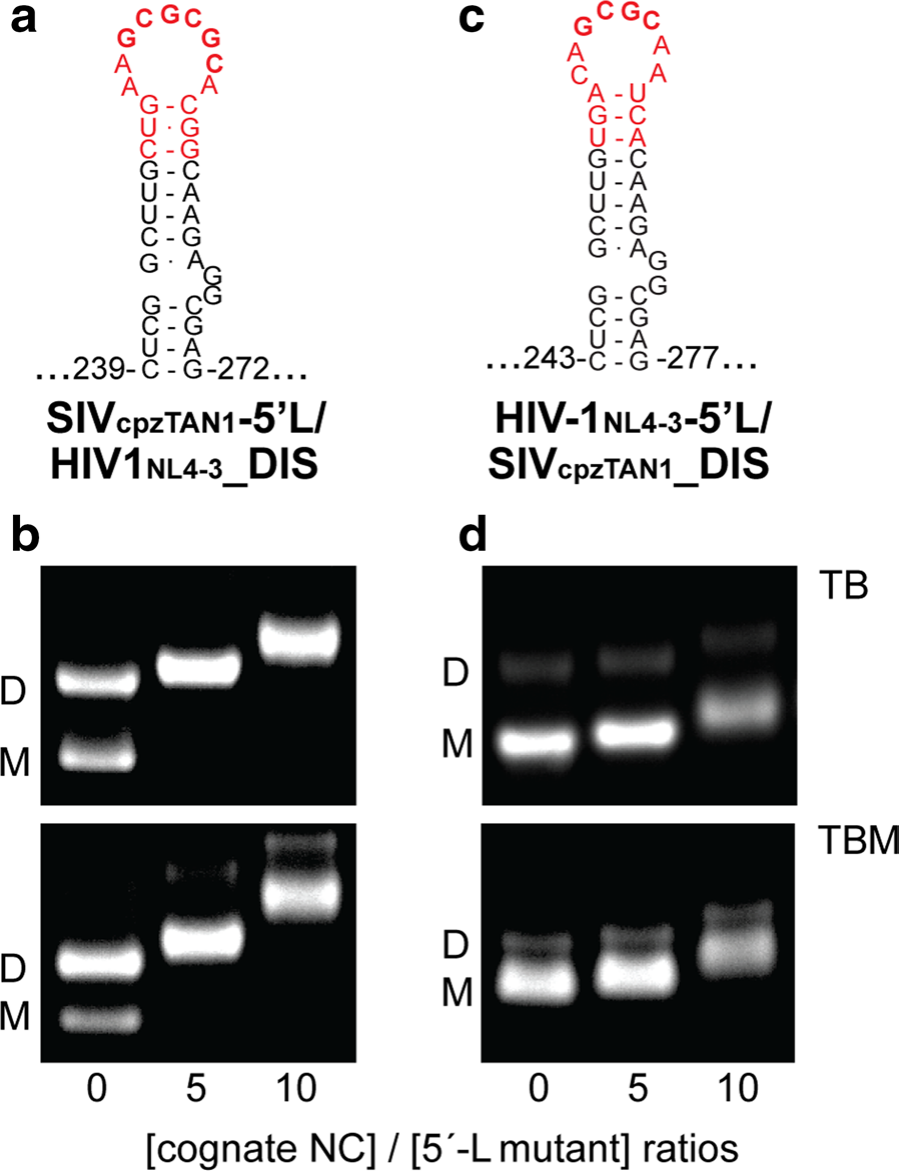
NC dependent dimerization is sensitive to the nature of the DIS loop. **a**, **c** Mutations in the loop and the stem of the DIS are colored red on the secondary structures of the SIV_cpzTAN1_ and HIV-1_NL4-3_ DIS within the intact leaders. Native gel NC titration results obtained for SIV_cpzTAN1_ RNA containing HIV-1 DIS loop (SIV_cpzTAN1_ 5′-L/HIV-1_NL4-3_ DIS) (**b**), and the HIV-1_NL4-3_ leader containing the DIS loop residues of SIV_cpzTAN1_ (HIV-1_NL4-3_ 5′-L/SIV_cpzTAN1_ DIS) (**d**) in PI buffer. Addition of 0, 5, 10 equivalents of NC to the respective retroviral mutants were probed using TB and TBM running buffers. Monomers (M) and dimers (D) are denoted. SIV_cpzTAN1_ NC binding induces a significant equilibrium shift toward the dimer of the SIV_cpzTAN1_ 5′-L/HIV-1_NL4-3_ DIS RNA, whereas HIV-1_NL4-3_ NC does not induce a detectable shift in the equilibrium of the HIV-1_NL4-3_ 5′-L/SIV_cpzTAN1_ DIS chimeric leader RNA

Interestingly, substitution of the DIS loop of the SIV_cpzTAN1_ 5′-leader RNA by the HIV-1_NL4-3_ DIS loop led to NC-dependent dimerization behavior similar to that observed for the native HIV-1_NL4-3_ leader, Fig. 9a, b. Conversely, substitution of the HIV-1_NL4-3_ DIS loop by the DIS loop from the SIV_cpzTAN1_ leader led to NC-dependent dimerization properties similar to those observed for the native SIV_cpzTAN1_ leader RNA, Fig. 9c, d. These findings indicate that both the intrinsic and NC-dependent dimerization properties are governed primarily by the DIS element.

## Conclusions

Although DIS and U5:AUG structures are phylogenetically conserved among all retroviruses [13, 27], the functions of these elements are not fully understood. There is considerable evidence that the dimer promoting GC-rich palindrome of the HIV-1 DIS hairpin is sequestered in the monomer and that formation of long range U5:AUG pairing promotes dimerization by allosterically exposing the DIS loop residues [2, 13, 33]. These structural changes enable the loop residues of DIS to form an intermolecular [GCGCGC]_2_ kissing interface [25, 68–74], and there is strong in vivo evidence in support of this mechanism [7, 75]. However, other studies have suggested that the DIS hairpins of the related HIV-2 and SIV lentiviruses do not play roles in HIV-2 and SIV genome dimerization [43] and that U5:AUG pairing inhibits, rather than promotes, dimerization of the HIV-2 5′ leader [43, 44]. Since a number of prior in vitro studies of HIV-2 dimerization were conducted with 5′-L fragments that terminated at, or prior to, residue 561, and thus could not form an AUG hairpin [42, 76], we examined the dimerization behavior of HIV-2_ROD_, 5′ leader RNA constructs analogous to those used in our previous HIV-1 leader studies [33]. In addition, to probe for unifying principles, we prepared and examined corresponding SIV_cpzUS_ and SIV_cpzTAN1_ 5′ leader RNAs, and tested the role of the DIS loop residues in dimerization.

One unanticipated observation was that inclusion of as few as three non-native cytosine residues at the 3′-end of the HIV-1 5′ leader had a measurable effect on both the rate of dimerization (~2-fold reduction) and position of the monomer–dimer equilibrium (favoring the monomer). The non-native cytosines present in our previously employed HIV-1 constructs (which enabled DNA plasmid digestion using the common SmaI restriction enzyme) likely altered and stabilized the AUG hairpin structure, thereby lowering the free energy of the monomer and reducing the rate of U5:AUG annealing. For this reason, all RNAs used in the present study terminated precisely at native 3′ residues and did not contain non-native 3′ extensions.

As observed by others, we were only able to detect dimer bands for the full-length HIV-2 and SIV 5′ leader RNAs when magnesium was included in the gel running buffers. Similar findings were reported previously for the HIV-1_MAL_ 5′ leader, which contains a native GGAUCC DIS loop palindrome [77]. The chimeric leader RNAs containing the HIV-1_NL4-3_ DIS loop also exhibited non-labile dimerization behavior. It thus appears that non-labile dimers are only formed by 5′ leader RNAs that contain a GCGCGC DIS loop sequence. In contrast, no dimers (labile or non-labile) were detected for the HIV-2_ROD_ mutant that lacks a palindromic DIS loop, and chimeric RNAs containing hybrid loops exhibit intrinsic and NC-dependent dimerization propensities (and labilities) similar to those of the native RNA from which the DIS loop sequence was derived. Thus, our findings do not support proposals for PBS or TAR mediated dimerization [43–46, 78], but are compatible with biochemical, mutagenesis, and genetic experiments indicating that the DIS hairpin is the primary determinant of genome dimerization [8, 11, 41, 47, 48, 79–81]. Notably, in studies involving co-transfection of cells with both HIV-1 and HIV-2, small amounts of virions containing native HIV-1:HIV-2 RNA heterodimers were observed, but heterodimer formation could be increased significantly by mutating the six-nucleotide DIS loops to enhance inter-viral RNA base pair complementarity and reduce intra-viral complementarity [11]. The low level of sequence complementarity outside of the engineered DIS palindromes makes it unlikely that other regions of the HIV-1 and HIV-2 5′ leaders participate directly in intermolecular dimer interactions [11].

The propensity to form labile dimers increases in the order: 5′-L_ΔAUG_ < 5′-L_WT_ ≤ 5′-L_LR_. This trend is observed for HIV-1_NL4-3_, SIV_cpzTAN1_, SIV_cpzUS_, and HIV-2_ROD_ 5′-L RNAs, and can be explained as follows. The 5′-L_ΔAUG_ construct, which lacks the entire AUG region, is unable to form U5:AUG base pairing, thereby favoring the monomeric species. In contrast, the 5′-L_LR_ contains all of the AUG residues necessary to base pair with U5, but lacks the downstream residues necessary to form an AUG hairpin structure that would compete with a U5:AUG structure. As such, the equilibrium adopted by 5′-L_LR_ is shifted more strongly toward the dimer. The AUG residues of 5′-L_WT_ can alternatively form a hairpin structure or base pair with U5, and as such, the monomer–dimer equilibrium of this wild-type RNA lies between that of 5′-L_ΔAUG_ and 5′-L_LR_, as observed for analogous HIV-1_NL4-3_ RNAs of previous studies [33].

Previous conclusions that U5:AUG base pairing inhibits HIV-2 RNA dimerization were based on observations made for non-labile dimeric species formed by shorter 5′ leader constructs that were unable to form an AUG hairpin. The facts that (1) the intact, wild-type HIV-2 and SIV leader RNAs readily adopts a monomer–dimer equilibrium that involves only the labile dimer upon incubation under physiological-like conditions (37 °C, PI buffer), and (2) significant amounts of non-labile dimers observed in prior studies were only detected for highly truncated RNAs, suggest that the non-labile dimers may be artifacts of truncation. Thus, residues near the 3′-end of the more highly truncated RNAs could be capable of interacting with upstream residues in a manner that exposes additional, unknown dimer-promoting residues (conceivably sites in the PBS loop) [82].

We also observed a marked pH-dependence of the monomer–dimer equilibrium for the SIV_cpz_ 5′ leader RNAs, whereas the HIV-1 and -2 retroviral leader RNAs did not exhibit significant sensitivity to pH under the conditions examined. Thus, adjusting the pH from 7.0 to 7.5 led to an equilibrium shift of the SIV_cpzUS_ leader from a predominantly monomeric to a predominantly dimeric species. Similarly, the SIV_cpzTAN1_ leader exhibited a large equilibrium shift towards the dimer over the same, relatively small pH range. Non-canonical A^+^·C wobble base pairs, in which the adenine N1 nitrogen is protonated and acts as a hydrogen bond donor [83], can have pKa values of ~7.0 or higher, and the differential presence of one or more A^+^·C wobble pairs in the monomer (but not the dimer) could explain the observed pH sensitivity. Previous studies have shown that protonation of adenosine A272 of the HIV-1 DIS loop enhances conformational dynamics of the loop–loop kissing species [84], but does not appear to detectably affect the position of the monomer–dimer equilibrium. Neither of the SIV leader RNAs are predicted to contain A^+^·C mismatches in the U5:AUG helix that might explain the pH sensitivity of the monomer–dimer equilibrium. However, both contain DIS loops with adenosines that could be differentially protonated in the dimer, and structural studies of the SIV DIS hairpin RNAs are warranted. Although some studies suggest that HIV infection can induce a small drop in cytoplasmic pH [85], we do not believe that the pH-dependent behavior observed here has a biological role. Instead, our findings illustrate the importance of accounting for pH when making in vitro biophysical comparisons.

**Fig. 10:**
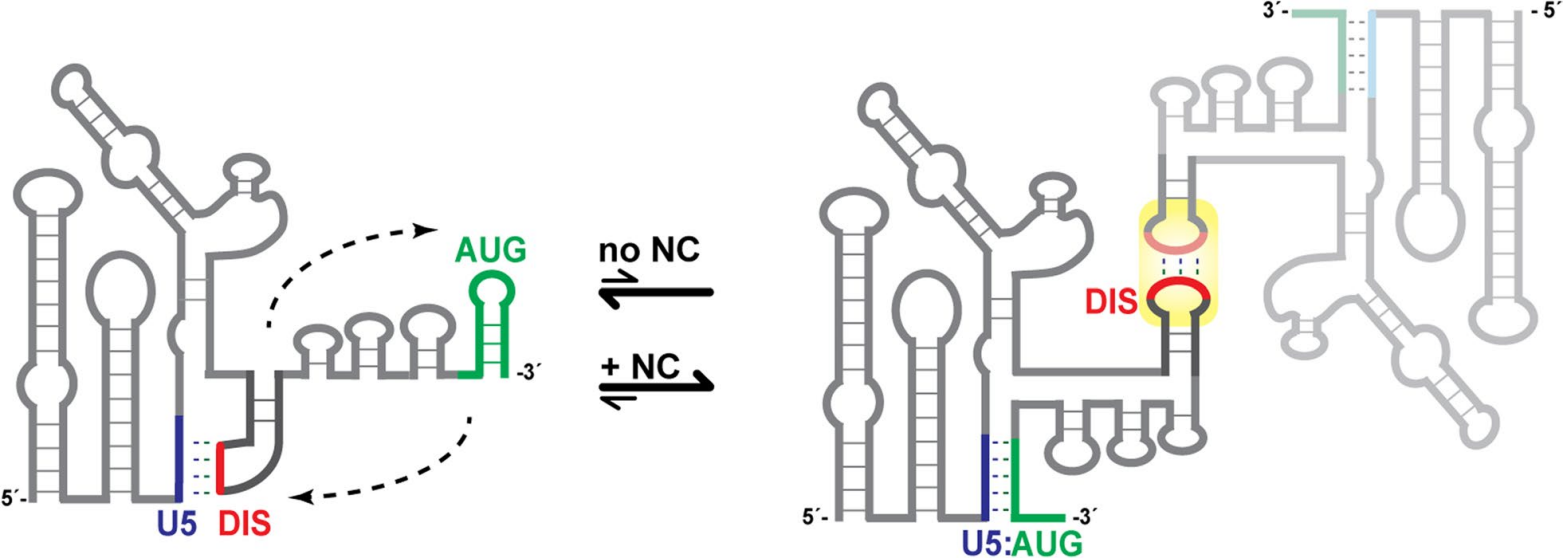
The proposed model for SIV_cpz_ and HIV-2 5′-leader dimerization mechanism. SIV_cpz_ and HIV-2 utilize a nucleotide displacement mechanism similar to HIV-1 [33] to regulate their dimeric genome packaging. In the presence of NC, retroviral 5′-leaders favor dimer containing U5:AUG formation

In summary, our findings support proposals for U5:AUG base pairing in the SIV_cpz_ and HIV-2 5′ leader RNAs and indicate that U5:AUG formation and NC binding promote dimerization (Fig. 10). Thus, the evolutionarily conserved DIS and U5:AUG elements appear to have common functions among these divergent lentiviruses. The intrinsic and NC-dependent dimerization propensities and labilities, which vary considerably among these lentiviral RNAs, appear to be established primarily by the composition of the DIS loop. The NC proteins exhibit cross-species dimer promoting abilities, but are unable to convert labile dimers to non-labile dimers. Although our studies suggest that the enhanced labilities of the HIV-1_MAL_, SIV_cpz_, and HIV-2_ROD_ dimers relative to those of HIV-1_NL4-3_ and HIV-1_LAI_ are due to the lower GC content of the DIS palindromes, it remains unclear to us why, or if, the significant kinetic differences would be evolutionarily advantageous to these different families of retroviruses. Of course, the in vitro RNA dimer labilities (as defined here and expressed as “loose dimers” in earlier work) reflect relative rates of dimer dissociation that occur when Mg^2+^ is stripped away during gel electrophoresis, and this is unlikely to occur in vivo since cells contain levels of Mg^2+^ and monovalent cations sufficient to maintain dimerization. It is thus possible that the biologically relevant dimer association equilibrium constants of the different retroviral RNAs may be similar. Efforts to test this hypothesis will require a new detection method that does not perturb the equilibrium (underway).

## Methods

### Plasmid construction for in vitro transcription

HIV-1_NL4-3_ 5′-L_WT_ plasmid with three additional C residues at 3′ end [33] were modified by mutagenesis to make native HIV-1_NL4-3_ 5′-L_WT_ for this study. HIV-1_NL4-3_ 5′-L_LR_ and HIV-1_NL4-3_ 5′-L_ΔAUG_ were subcloned from the native HIV-1_NL4-3_ 5′-L_WT_ plasmid. SIV_cpzTAN1_ 5′-L_WT_ was amplified from the SIVcpzTAN1.910 clone (obtained through the NIH AIDS Reagent Program, Division of AIDS, NIAID, NIH, from Drs. Jun Takehisa, Matthias H. Kraus and Beatrice H. Hahn, GenBank EF394356). SIV_cpzTAN1_ 5′-L_ΔAUG_ and 5′-L_LR_ were subcloned from SIV_cpzTAN1_ 5′-L_WT_. SIV_cpzUS_ 5′-L_WT_ DNA sequence was ordered from Genewiz. SIV_cpzUS_ 5′-L_ΔAUG_ and 5′-L_LR_ were subcloned from SIV_cpzUS_ 5′-L_WT_. HIV-2 5′-L_ΔAUG_ and 5′-L_LR_ were subcloned from HIV-2 5′-L_WT_ plasmid. All of the forward primers (Integrated DNA Technologies) used to clone HIV-1, SIV_cpzTAN1_, SIV_cpzUS_, and HIV-2_ROD_ native and mutant fragments are listed in Table 1. The QuikChange Lightning Site-Directed Mutagenesis protocol (Agilent) was used for the mutagenesis process. All of the DNA plasmids were sent to Genewiz sequencing to ensure that the desired mutations were obtained.

**Table 1:** DNA primers for plasmid constructions

Name	DNA sequence
HIV-1_NL4-3_	
5′-L_ΔAUG_	5′-TGA CTA GCG GAG GCT AGT ACT AGA GAG ATG GGT GCG-3′
SIV_cpzTAN1_	
5′-L_WT_	5′-GAG AGC GTC AGT GTT GCA GGG GGA TCC TCT-3′
5′-L_ΔAUG_	5′-GTC TCT AGG TAA CAG CTG AAA TGG GTG CGA G-3′
5′-L_LR_	5′-GGA AAT GGG TGC GAT ATC GTC AGT GTT-3′
5′-L_GC_	5′-TCG GCT TGT GAC AGC **GC**G CAA TCA CAA GAG GCG A-3′
5′-L_HIV-1_loop_	5′-GCA GGA CTC GGC TTG TGA **AAG CGC GCA** TCA CAA GAG GCG AGG CGG-3′
NC	5′-CGG GCC ATA TGC TGC AGG GAG GAA AAA GA-3′
NC	^a^5′-GCG GAG GAT CCT CAG TTG TTT CTG GTG GTG CAG TC-3′
SIV_cpzUS_	
5′-L_WT_	^a^5′-CCG TCG GAT CCG TTA ACT GAC GCT CTC GCA CCC AT-3′
5′-L_ΔAUG_	5′-GTC TCT AGG GGA AGG CCA TGG GTG CGA TAT C-3′
5′-L_LR_	^a^5′-CCG TCG GAT CCG ATA TCG CAC CCA TCC TCT TCC CCT-3′
NC	5′-CGG GCC ATA TGC TAC AGA AAG GAA ACG CT-3′
NC	^a^5′-GCG GAG GAT CCT CAA TTA GCC TGT CTG TTT CC-3′
HIV-2_ROD_	
5′-L_WT_	5′-CGA GA AAC TCC GTC TTG CA CCG GGG GAT CC TCT AGA-3′
5′-L_ΔAUG_	5′-TTT AGA CAG GTA GAA GAT ATC GGG AGA TGG GCG CGA GAA-3′
5′-L_LR_	5′-GGG AGA TGG GCG CGA TATC ACT CCG TCT TGA CCC-3′
5′-L_CCA_	5′-AAA GGC GCG GGC CGA **CCA** ACC AAA GGC AGC GUG-3′
5′-L_HIV-1_loop_	5′-AGA AAG GCG CGG GCC **AAG CGC GCA** GGC AGC GTG TGG AGC-3′
5′-L_GAGA_	5′-AGA AAG GCG CGG GCC **GAG A**GG CAG CGT GTG GA-3′

^a^Reverse primers

Recognition sites for restriction endonucleases are underlined. Introduced mutations are bolded

### RNA synthesis and purification

pUC19 plasmids carrying different RNA clones were amplified in *E. coli* XL10-Gold ultracompetent cells. Large-scale DNA plasmid preparation was performed using QIAGEN Plasmid Mega Kit (Qiagen). Each plasmid was linearized using a specific restriction enzyme (Table 1 with underlined recognition sites for each RNA construct). Each in vitro RNA transcription by T7 RNA polymerase [86] was performed in solution with a mixture of transcription buffer, MgCl_2_, NTPs, and a corresponding linearized DNA template [52]. RNAs were then purified in 5 % (HIV-2 RNA constructs) and 6 % (HIV-1 and SIV_cpz_ RNA constructs) denaturing PAGE gels (National Diagnostics) and extracted using Elutrap Electroelution system (Whatman). The final RNA product was obtained after two 2 M NaCl washes and eight ddH_2_O washes using Amicon ultra centrifugal filter units (Millipore).

### NC purification

The 55-residue HIV-1_NL4-3_ NC was expressed and purified as previously described [52, 87]. The native DNA sequence coding for the 56-residue SIV_cpzTAN1_ NC was subcloned from the full length SIVcpz.910 clone [88]. The DNA sequence coding for the 58-residue SIV_cpzUS_ NC was designed and ordered from Genewiz. Subsequently, both SIV_cpzTAN1_ and SIV_cpzUS_ NC DNA sequences were cloned into pET-11a expression vectors (Novagen) and transformed into Rosetta(DE3) and Rosetta(DE3)pLysS cell lines, respectively, for protein expression. The desired forward and reverse primers with additional restriction sites used for SIV_cpz_ NC cloning are listed in Table 1. The proteins were purified in a similar approach as previously described [87]. The final protein products were in physiological-like (PI) buffer (10 mM Tris, 140 mM KCl, 10 mM NaCl, 1 mM MgCl_2_, pH 7.0) with 1 mM TCEP and stored at −80 °C.

The gene coding for the 49-residue HIV-2_ROD_ NC was inserted into the pGEX-6P-1 vector (a gift from Dr. Barry Johnson) and expressed in BL21(DE3) cells. Cells were harvested after 4 h of IPTG-induced protein over-expression and lysed in phosphate buffered saline (PBS) buffer with 0.5 mM DTT. After 4 % (w/v) polyethyleneimine (PEI) precipitation, supernatant containing the target protein was filtered with 0.45 μm filter and applied to pre-equilibrated glutathione resin (Genscript). The supernatant and resin were shaken at 4 °C overnight. After draining the column, the resin was washed with 150 mL of PBS buffer (0.5 mM DTT) and 150 mL cleavage buffer (50 mM Tris, 150 mM NaCl, 0.5 mM DTT, pH 7.8). PreScission Protease (GE) in 25 mL pre-cooled cleavage buffer was added to the resin and shaken at 4 °C overnight. The elution was incubated with 5 mM DTT for 1 h at room temperature, then dialyzed into refolding buffer (10 mM Tris, 5 mM NaCl, 0.3 mM ZnCl_2_, 50 mM l-Arginine, 50 mM l-Glutamic Acid, 0.1 mM 2-Mercaptoethanol, pH 7.3) overnight. HIV-2_ROD_ NC protein was then concentrated and dialyzed in PI buffer with 0.1 mM BME. The integrity of the SIV_cpz_ and HIV-2 NC proteins were confirmed by mass spectrometry.

### RNA dimerization assay

HIV-1_NL4-3_ and HIV-2_ROD_ RNAs were boiled in ddH_2_O for 3 min and snap cooled on ice for 2 min. HIV-1_NL4-3_ 5′-L_ΔAUG_ was boiled in a mixture of 10 mM Tris–HCl, pH 7.0, 10 mM NaCl, and 140 mM KCl for 3 min, snap cooled on ice for 2 min, with 1 mM MgCl_2_ added prior to incubation (Note: for this sample only, boiling/snap cooling in water alone resulted in misfolding and formation of higher order species. Higher order oligomers were not observed for any of the RNA samples were boiled/cooled in Tris buffer with monovalent ions). Experiments for HIV-1_NL4-3_ and HIV-2_ROD_ RNAs conducted in the absence of boiling and snap cooling gave similar results. RNA samples for HIV-1_NL4-3_, SIV_cpzTAN1_, SIV_cpzUS_, and HIV-2_ROD_ were prepared at 0.9 μM in PI buffer and incubated at 37 °C for various intervals. After the incubation, samples were loaded into an ethidium bromide pre-stained native agarose gel (1 % for HIV-1_NL4-3_, SIV_cpzTAN1_, and SIV_cpzUS_, 0.8 % for HIV-2_ROD_) and electrophoresed at 4 °C in TB condition (44 mM Tris–borate, pH 8.3) or TBM condition (44 mM Tris–borate, pH 8.3 with additional 0.2 mM MgCl_2_ for HIV-1_NL4-3_, SIV_cpzTAN1_, SIV_cpzUS_, and 0.25 mM MgCl_2_ for HIV-2_ROD_) at 15 V/cm.

### NC:RNA binding assay

HIV-1_NL4-3_, SIV_cpzTAN1_, SIV_cpzUS_, and HIV-2_ROD_ RNAs were prepared as described above, then mixed with various molar ratios (0, 5, 10) of corresponding NC protein in PI buffer. The NC-RNA mixtures were incubated at 37 °C for 1 h before analysis by native agarose gel electrophoresis (4 °C; 10.5 V/cm; 65 min electrophoresis time) under both TB and TBM running conditions.

## Abbreviations

HIV-1_NL4-3_: human immunodeficiency virus Type-1 NL4-3 strain
SIV_cpz_: simian immunodeficiency virus in chimpanzee
SIV_cpzTAN1_: simian immunodeficiency virus obtained from a wild-caught chimpanzee in Tanzania
SIV_cpzUS_: simian immunodeficiency virus obtained from a wild-caught in Africa and raised in United States
HIV-2_ROD_: human immunodeficiency virus Type-2 ROD strain
NC: nucleocapsid
5′-L: 5′ leader
U5: unique 5′ element
DIS: dimerization initiation site
AUG: stem-loop containing AUG start codon for the translation of *gag*
